# Editorial: The immunological events of macrophages in the course of sepsis

**DOI:** 10.3389/fimmu.2025.1610868

**Published:** 2025-05-01

**Authors:** Shiyun Wu, Xu Li

**Affiliations:** Department of Emergency Medicine, Nanfang Hospital, Southern Medical University, Guangzhou, China

**Keywords:** macrophages, sepsis, cytokine, metabolite, Mendelian randomization

Sepsis is a complex disease with high morbidity and mortality. It remains a significant cause of mortality on a global scale. According to the third international consensus definition of sepsis and septic shock (Sepsis-3), sepsis is defined as life-threatening organ dysfunction due to a dysregulated host response to infection (1). However, the Sepsis-3 definition of sepsis underscores the significance of organ dysfunction, while its pathophysiological processes remain to be fully elucidated. Despite the paucity of research in this area, scholars worldwide have conducted extensive research on the pathophysiological mechanisms of sepsis, and mounting evidence supports the central role of the immune system in the development of sepsis. Specifically, the host’s innate and inflammatory response to infection is dysregulated (2).

Macrophages, a critical component of the innate immune system, are the predominant immune cells present in all organs (3, 4) These cells exhibit significant heterogeneity and phenotypic specialization, which are regulated in a tissue-specific manner. The identification of alterations in specific macrophage subpopulations and their correlation with disease progression is a pivotal approach to comprehending the pathophysiologic progression of sepsis and to identifying therapeutic targets. The presence of tissue-resident macrophages (R-MACs) enables them to promptly respond to local injury (5). These cells are the most abundant immune cells in many tissues, and they are likely to play an extremely important role in the distant or even systemic immune status.

**Figure 1:**
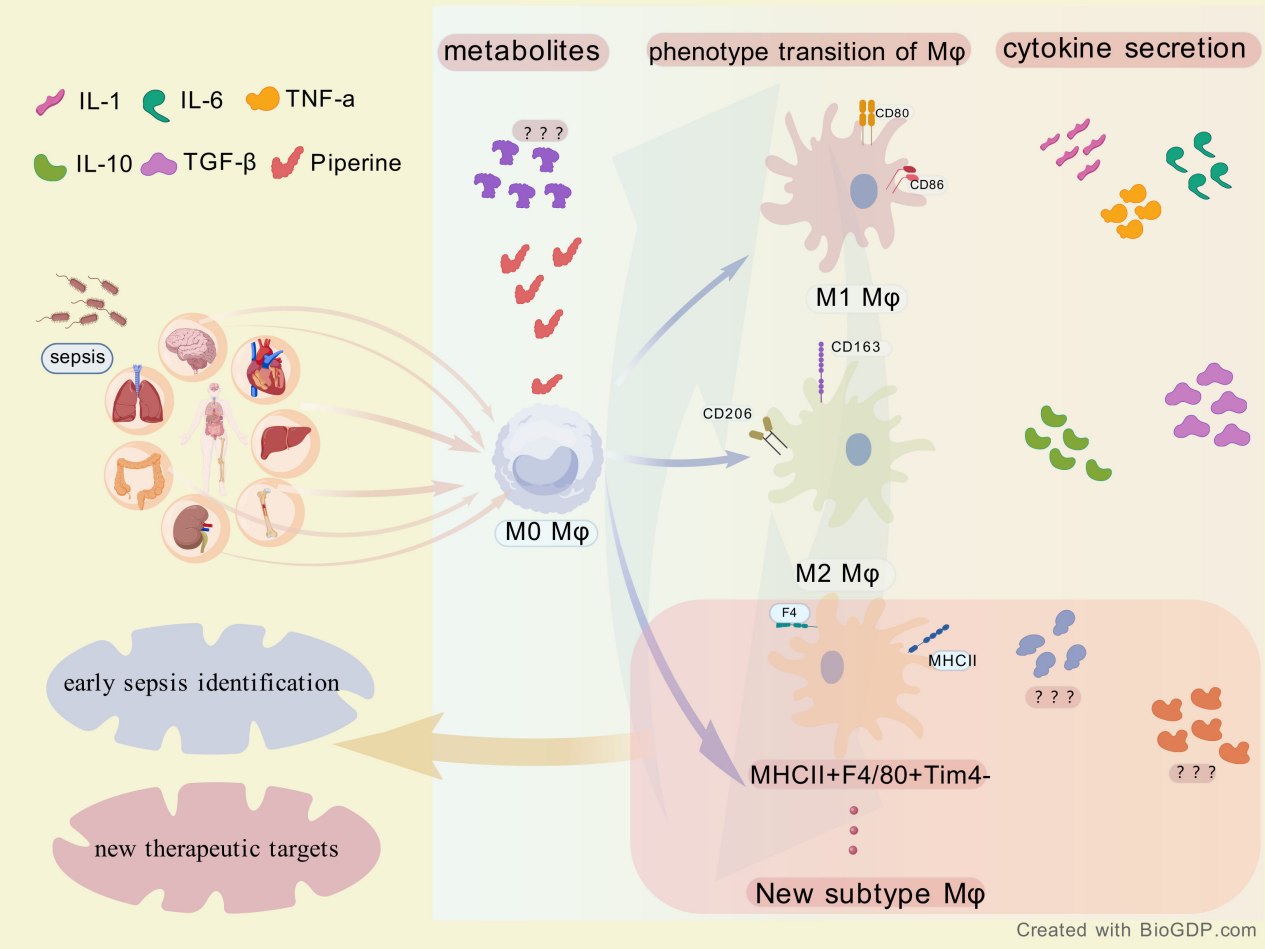
The association between sepsis and macrophages (Mφ). Sepsis affects multiple organs and acts on M0 macrophages in their initial state. M0 macrophages can differentiate into M1 and M2 macrophages as well as a new subtype (MHCII + F4/80 + Tim4 - ), accompanied by alterations in metabolites and secretion of cytokines such as IL - 1 and TNF - α. This process provides insights for the early identification of sepsis and the exploration of new therapeutic targets.

Furthermore, macrophages have been shown to play a pivotal role in the inflammatory pathway by recognizing pathogens and damage-associated molecules and secreting EVs. The chemokine, CXCL2, found in EVs, has been demonstrated to play a crucial role in recruiting neutrophils to the site of infection (6) and to regulate the inflammatory pathway by predicting and modifying miRNAs in EVs (7). The question of interest is what other mechanisms through which macrophages influence sepsis disease changes as well as disease regression throughout the course of sepsis. The alterations in sepsis that result from inflammatory factor release, phenotypic changes, or cell death of resident macrophages in different organs have also been a subject of interest (**Figure 1**).

Khanna et al. identified MHCII^+^F4/80^+^Tim4^-^ macrophages as a significant contributor to the pro-inflammatory milieu in cytokine storm syndrome (CSS) from the perspective of altered hepatic macrophage phenotypes and their M1/M2 subpopulation ratios with disease progression, emphasizing the importance of macrophage-targeted interventions in the effective management of CSS and sepsis. Shimizu et al. They also initiated research on liver-resident Kuffer cells and explored inhibitors targeting eCIRP for the purpose of regulating phenotypic changes in Kuffer cells by studying the molecular mechanism of extracellular cold-inducible RNA-binding protein (eCIRP), which acts as a damage-associated molecular pattern (DAMP) to directly alter Kuffer cell phenotype and function. Zheng et al. and Gong et al. employed a Mendelian randomization (MR) approach, focusing on metabolites, inflammatory factors, and sepsis. They investigated the causal relationship between metabolites, immune phenotype, and sepsis, respectively. These metabolites and inflammatory factors may play important roles in the pathophysiology of sepsis and may serve as potential therapeutic targets or biomarkers for sepsis management. Notably, Zheng et al. discovered that the proportion of CD14^+^ CD16^+^ monocytes in the total monocyte population is a pivotal factor in sepsis outcomes, indicating a direct association with disease severity. In addition, Gong et al. reported findings of particular interest regarding the impact of 1400 metabolites on sepsis, including the initial observation that piperine exerts an effect on the progression of sepsis and confers protection against sepsis by influencing AXIN1.

The knowledge gained from these studies not only deepens our understanding of the immunopathology of sepsis, but also holds promise for the development of innovative therapies. For example, studies by Khanna et al. and Shimizu et al. suggest that therapies may be designed to manipulate macrophage polarization to a more favorable state or to target specific cytokine pathways to optimize the immune response. In turn, the relevant inflammatory factors found by Zheng et al. and Gong et al. through MR can guide us to molecular mechanisms and pathways in the sepsis process, so that we can find therapeutic targets for sepsis.

In conclusion, the articles in this theme provide a partial overview of the immunological events in macrophages during sepsis. Filling these knowledge gaps will not only deepen our understanding of the pathophysiological mechanisms of sepsis and provide valuable insights into current challenges and advances in the field. It may also lead to many innovative therapeutic approaches targeting sepsis starting from macrophages. It must be acknowledged that this article exhibits certain deficiencies. These include the potential presence of residual confounders resulting from chain imbalance or multi-effectiveness (pleiotropy) in the MR category. This engenders skepticism regarding the article’s credibility. Nevertheless, it is anticipated that the article will stimulate additional research in this domain and, consequently, contribute to the enhancement of the clinical prognosis of patients afflicted with sepsis.
